# On the remarkable thermal stability of nanocrystalline cobalt via alloying

**DOI:** 10.1016/j.msea.2014.11.062

**Published:** 2015-01-29

**Authors:** A. Bachmaier, C. Motz

**Affiliations:** Chair of Materials Science and Methods, Saarland University, Saarbrücken, Germany

**Keywords:** Nanostructured materials, Severe plastic deformation, Thermal stability, Cobalt

## Abstract

Nanostructured Co materials are produced by severe plastic deformation via alloying with small amounts of C and larger amounts of Cu. The thermal stability of the different nanostructured Co materials is studied through isothermal annealing at different temperatures for various times and compared to the stability of severe plastically deformed high-purity nanocrystalline Co. The microstructural changes taking place during annealing are evaluated by scanning electron microscopy, transmission electron microscopy and microhardness measurements. In the present work it is shown that the least stable nanostructured material is the single-phase high purity Co. Alloying with C improves the thermal stability to a certain extent. A remarkable thermal stability is achieved by alloying Co with Cu resulting in stabilized nanostructures even after annealing for long times at high temperatures. The essential reason for the enhanced thermal stability is to be found in the immiscibility of both components of the alloy.

## 1 Introduction

Nanocrystalline (nc) materials possess unique mechanical and functional properties, which are often enhanced or even completely different from the ones of their coarse grained counterparts [1]. Nc Co and Co alloys exhibit, for example, interesting magnetic properties like an extraordinary high saturation magnetization and the giant magneto resistance effect making them to one of the promising candidates for components in microelectromechanical systems (MEMS) [2,3]. Because their unique properties are manifested by their small grain size, one major reason restricting potential technological applications of nc structures is their low thermal stability. Another major reason is that the fabrication of massive, nc bulk materials in reasonable volumes, which would be necessary for future technical applications, is still a challenging task.

In this study, stable Co nanocrystallites are synthesized by severe plastic deformation (SPD) and different approaches to enhance the thermal stability of nc Co materials are compared. Only a few studies are currently available on SPD deformation of pure Co and Co alloys, where mainly the phase transformations induced by SPD are investigated [4,5]. Co exhibits a martensitic phase (ε phase with a close packed hexagonal (hcp) structure) at temperatures below ~695 K under equilibrium conditions. Above this transformation temperature, a α phase with a face-centered cubic (fcc) structure exists [6].

Nc materials are usually thermally unstable because of their large amount of enthalpy stored in the large grain boundary area. The driving force for grain growth scales inversely proportional to the grain size considering ideal grain boundary curvature-driven growth [7,8]. However, the thermal stability can be significantly improved if grain boundary migration is hindered. Several studies investigate the thermal stability of electrodeposited (ED) pure Co and Co-based nc materials [9,10]. For instance, grain growth in ED nc pure Co starts at temperatures already at 423 K [9]. Although alloying improves the thermal stability, abnormal grain growth still occurs even after annealing for only 1800 s at 573 K in an ED nc Ni–74%Co alloy. After 7200 s at the same annealing temperature, the nc matrix is completely consumed [10]. Sulfur, among other elements, is often used as additive during the ED process to obtain nc metals. Sulfur acts as grain growth inhibitor during the deposition process itself and furthermore enhances the thermal stability. Nevertheless, abnormal grain growth is often observed because these grain growth inhibitors are not evenly distributed [11]. Hence, attempts to improve the thermal stability of nanostructures must go hand in hand with preventing grain growth.

In [12], the effect of the starting microstructure (different grain sizes in the as-deposited state combined with various impurity contents from additives) on the thermal stability of ED Co is studied, which is mainly governed by the total amount of sulfur due to a dynamic segregation mechanism. Although the thermal stability could be improved, significant grain growth yields to micrometer-sized grains for temperatures as low as 773–873 K [12]. Another disadvantage emerges from the use of additives during the ED process. Their individual non-metallic constituents diffuse to grain boundaries even at moderate annealing temperatures due to their low solubility in the ED metals. In [13] it is shown that even very small amounts of sulfur (50 ppm) have a detrimental effect on the mechanical properties of pure ED Cu due to grain boundary embrittlement. The loss of ductility due to grain boundary embrittlement must be considered for future technological applications of nc Co and Co alloys, e.g. for MEMS applications, as well. Another element, which might be used as effective grain growth inhibitor in nc Co, is carbon. Due to its small size and its low solubility in Co, segregation to interfaces like grain boundaries can be expected. Adding carbon to pure Ni leads to high-strength nc Ni with a reasonable ductility for this strength regime after SPD which is unaffected by the carbon content [14].

In this study, two approaches to enhance the thermal stability of nc Co and Co alloys are used. Firstly, nanostructured materials of high-purity Co and high-purity Co doped with a small amount of carbon are generated by high-pressure torsion (HPT) to investigate the influence of carbon on thermal stability. One major advantage of HPT is that nc materials with reasonable dimensions can be produced and second phase particles (e.g. carbon) can be homogeneously distributed in the corresponding metal matrix [15]. The second approach used in this study to enhance the thermal stability of nc Co is based on alloying, whereby Cu is chosen as alloying element. The binary Co–Cu system is immiscible at room temperature. Although there exists no equilibrium solubility, the formation of non-equilibrium solid solutions has already been observed in this alloy system during ball milling at ambient temperature [16–21]. The formation of non-equilibrium solid solutions can also be obtained by HPT deformation in different immiscible systems. During annealing of the formed solid solutions, decomposition and the formation of a two phase structure is observed [22,23]. Therefore, nc Co-based Cu alloys by HPT deformation of powder compacts are generated from commercially available micrometer sized Co and Cu powders to evaluate possible formation of supersaturated solid solutions by HPT deformation and its effect on thermal stability. By the use of powders with different purity levels, the impurity amount is also adjusted.

The thermal stability of the nc structures obtained by the two different approaches is investigated and compared to HPT deformed high-purity Co to explain their effect on the thermal stability. Special attention is given to the influence of allotropic phase transformation, the effect of alloying and the possible formation of supersaturated solid solutions in the alloy on thermal stability. These effects are furthermore discussed together with the observation of small pores in the nc Co-based Cu alloy samples.

## 2 Experimental

In this study, pure Co and Co-based Cu alloys of different initial conditions (powder and bulk material) are processed by HPT. Commercial Co with a high-purity (Puratronic^®^ from Alfa Aesar) and Co of the same high-purity material doped with a small amount of carbon (500 ppm) are investigated. The type and total amount of included impurity elements from the materials analysis provided by the supplier of the high-purity Co material are listed in Table 1. Only elements with values ≥0.1 ppm are given in the table. To produce the carbon-doped samples, the corresponding graphite mass is arc melted together with the Co material. To guarantee a homogeneous carbon distribution in the sample, the arc-melting process is repeated five times. Afterwards the carbon content is determined by the analytical services Goslar (H.C. Starck GmbH) by carbon combustion analysis. A total carbon amount of 457 ppm is determined in the material after arc-melting. From the above mentioned Co materials, disk shaped samples with a diameter of 8 mm and a thickness of 0.8 mm are HPT deformed at room temperature for 10 rotations. The pressure during the HPT deformation is 6.0 GPa. The rotation speed is kept constant at 0.2 rpm. The aforementioned processed material is denoted as ‘pure Co’ and ‘doped Co–C’ in this study.

**Fig. 1:**
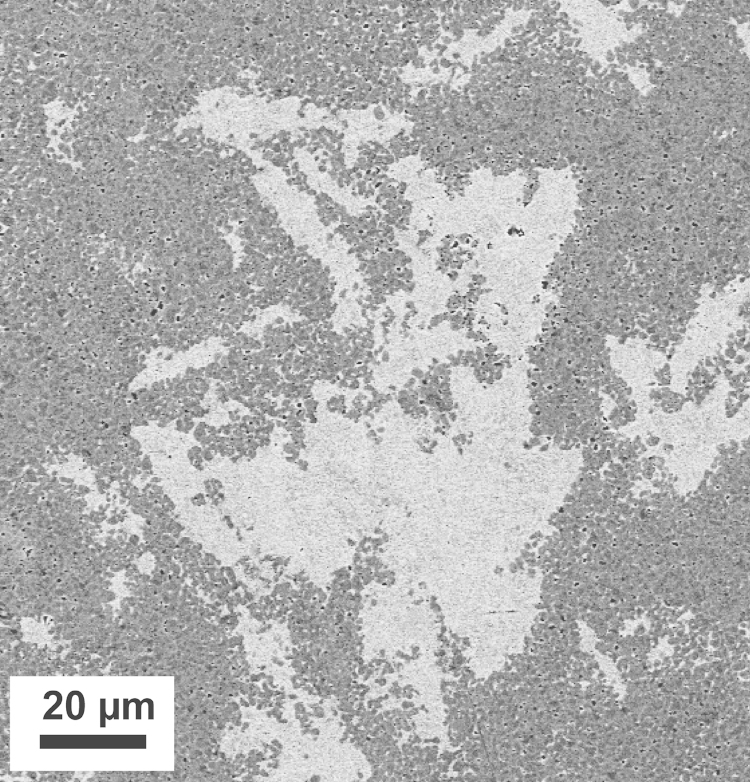
SEM image (back scattered electron mode) of the microstructure of the Co75Cu25 sample in the as-fabricated, undeformed condition.

Furthermore, a two-phase Co–Cu alloy fabricated by RHP-Technology (Seibersdorf, Austria) is processed by HPT. Co powders (purity 99.8%) and Cu powders (purity 99.9%) are mixed containing 25 and 75 weight-percent (wt%) Cu and Co, respectively. For comparison and to study the influence of the impurity content, Co and Cu powders with a higher purity (99.9 and 99.99%) are additionally mixed in the same composition and subsequently hot-isostatically pressed at 900 °C under vacuum into samples with cylindrical shape (diameter of 20 mm and a thickness of 11 mm). The initial microstructure in the as-fabricated condition consists of a Co matrix with Cu particles (Fig. 1). The size of the Cu particles is strongly varying between 20 and 150 µm. Initial grain size in the Cu particles is between 0.2 and 1.0 µm, the grain size in the Co matrix is significantly smaller (~0.1 to 0.2 µm) [24]. From the as-fabricated material, rods with a diameter of 8 mm are turned, cut into disks with a thickness of 0.6 mm and HPT deformed at room temperature (pressure of 5.0 GPa, 25 rotations, rotation speed 0.2 rpm). In this study, the above described material is denoted as ‘Co75Cu25-L’ and ‘Co75Cu25-H’, in which L and H denotes the material with low and high purity, respectively. A detailed description of the HPT equipment used in this study and a general overview about the HPT deformation process are given in [25,26].

After HPT deformation, all samples are subjected to isothermal heat treatments at 573 K, 673 K and 873 K for 1 h at air to investigate the thermal stability of the deformed material. Selected samples are also annealed at 423 K, 573 K, 673 K and 873 K for 1 h, 4 h, 7 h, 27 h and 100 h to investigate the long term stability of the processed materials. The microstructures of the materials are characterized by scanning electron microscopy (SEM) using a ZEISS SIGMA VP device and by transmission electron microscopy (TEM) using a JEOL JEM 2010 instrument operated at 200 kV. Electron-back scatter diffraction (EBSD) is conducted to identify grains and phases in the annealed conditions using the EBSD system (Oxford Nordlys EBSD detector) attached to the SEM and Oxford HKL Channel 5 analysis software. Orientation and phase information is acquired using different step sizes (20–150 nm) dependent on scan and grain size. All micrographs and EBSD scans are taken at a fixed radius of 3.5 mm of the HPT disks in this study. A radius of 3.5 mm corresponds to a *ε*_*v*_ of about 160 (10 rotations, pure and doped Co samples) and to a *ε*_*v*_ of about 530 (25 rotations, Co75Cu25 samples). The TEM sample preparation includes the following steps: disks were cut at a radius of 2.5 mm of deformed and selected annealed samples (pure and doped Co samples and Co75Cu25 sample), mechanically thinned and polished to a thickness of about 100 μm. Afterwards, mechanical dimpling until the thinnest part reaches a thickness of about 10 μm is conducted. The samples are subsequently ion-milled with Ar ions at 4–5 kV under an incidence angle of 5–7° using a Gatan Precision Ion Polishing System until perforation was obtained. X-ray diffraction (XRD) analysis is performed on deformed and selected annealed material using Cu-Kα radiation (PANalytical X’pert diffractometer in *θ*/2*θ*-geometry). Vickers microhardness measurements are conducted using a load of 500 g (HV0.5) across the radii of the disks (3 indents at each position) with a spacing of 0.25 mm between the individual indents in the as-deformed and annealed conditions.

## 3 Results

### 3.1 Characterization of as-deformed nanostructures

**Fig. 2:**
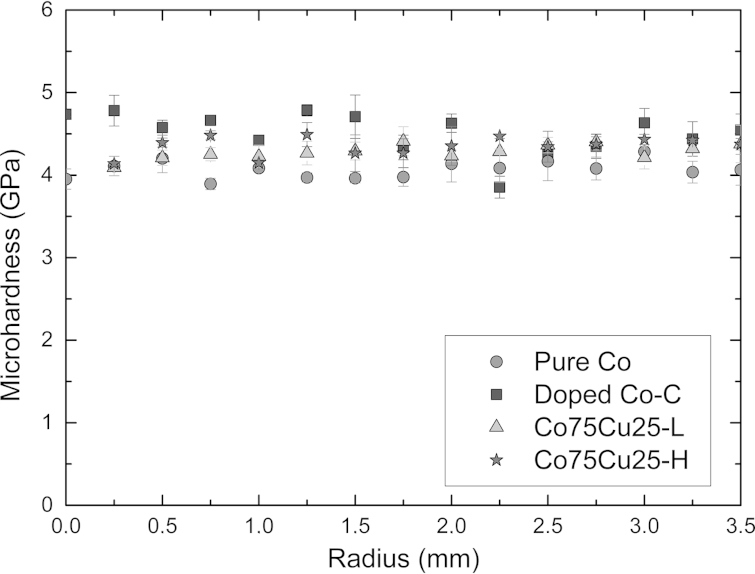
Microhardness profiles for all three samples along the HPT-disc radius deformed to 10 rotations (pure Co, doped Co–C) and 25 rotations (Co75Cu25-L and Cu75Co25-H). All three samples show the saturation regime.

**Fig. 3:**
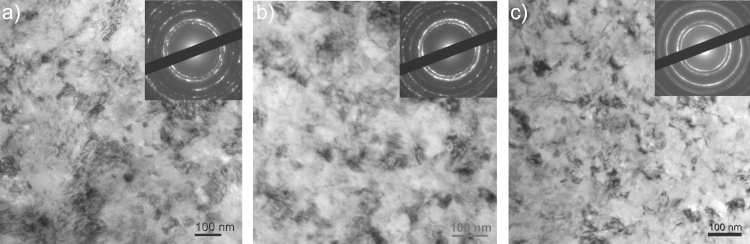
TEM bright field images with corresponding SAD patterns (see insets) of the microstructure of the samples in the as-deformed condition in the saturation regime: (a) pure Co, (b) doped Co–C and (c) Co75Cu25-L. Please note the difference in magnification.

Fig. 2 shows the microhardness data of the as-deformed samples. The different Co materials exhibit a rather constant microhardness as a function of the radii of the HPT samples and the typical microhardness distribution of HPT samples, which are only partly deformed to saturation, is not observed [27]. Therefore, saturation of the microstructural refinement – except the very near center – is reached in all deformed materials and application of further HPT deformation will not induce further grain refinement [25]. Mean values of the microhardness in the as-deformed condition of the different samples are listed in Table 2. As-deformed pure Co exhibits a microhardness of 4.07±0.11 GPa, doped Co–C a microhardness of 4.52±0.25 GPa and the Co75Cu25-L and Co75Cu25-H alloyed samples a microhardness of 4.28±0.04 GPa and 4.35±0.11 GPa, respectively. Fig. 3 presents the microstructure (bright field TEM images) of the samples in the as-deformed condition. All samples show the same type of uniform ultra-fine grained or nc microstructures. Contrast variations inside the grains, which indicate a huge amount of defects in the as-deformed structure makes a clear identification of the grains and grain boundaries difficult. Qualitatively, the largest grain size is obtained in the pure Co sample, which is in the ultra-fine grained regime (Fig. 3a). The doped Co–C sample exhibits a more refined microstructure with a grain size <100 nm (Fig. 3b). It is shown in [14], that carbon is the most important impurity element in controlling the limit of grain refinement for HPT-deformed Ni samples at room temperature. In our pure Co samples, a small amount of 500 ppm carbon has also a distinct effect on the final achievable grain size and microhardness in HPT deformed pure Co. In the Co75Cu25 samples, the smallest grain size in the as-deformed condition is obtained. The Co75Cu25-L sample exhibits a grain size only about half the size of the HPT deformed pure Co sample (Fig. 3c). In the Co75Cu25-H sample, a similar type of nanostructure with a slightly higher microhardness (4.35±0.11 GPa) is obtained. In the selected area diffraction (SAD) pattern shown in the insets in Fig. 3a and b, hcp Co Debye–Scherrer rings of (100), (002), (101), (102) and (110) for pure and doped Co samples are observed. For the Co75Cu25-L sample, fcc Co Debye–Scherrer rings of (111), (200), (220), (311) and (222) are visible (Fig. 3c). The observed strong grain refinement of the Co75Cu25-L sample is also reflected in the SAD pattern. In the SAD patterns of the pure and doped Co sample (see inset in Fig. 3a and b) single spots are still visible in the rings due to relatively large grains. In comparison, the SAD pattern of the Co75Cu25-L sample (see insets in Fig. 3c) demonstrates the nc grain size.

**Fig. 4:**
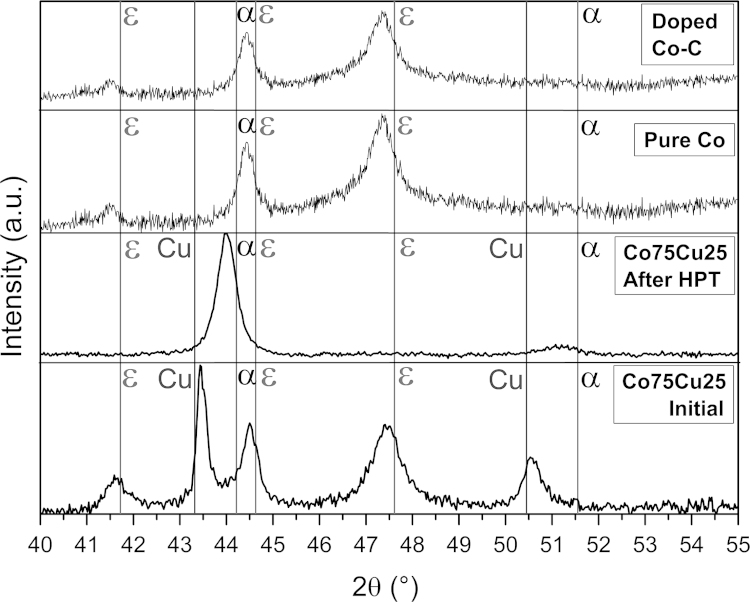
XRD patterns of the samples (pure Co, doped Co–C, Co75Cu25-L) in the as-deformed condition. The XRD pattern of the Co75Cu25-L sample in the as-fabricated condition is plotted at the bottom.

Fig. 4a illustrates the XRD patterns for the samples in the as-deformed condition. For the Co75Cu25-L sample, the XRD pattern of the as-fabricated (undeformed) state is additionally plotted. Generally, no peaks from oxides or other contaminants are observed in all recorded XRD patterns. The pure Co and doped Co–C samples exhibit only ε Co, while α Co cannot be detected in the XRD pattern in the as-deformed condition, which is consistent with TEM observations. This observation fit quite well to data already reported in previous studies for the crystal structures after HPT deformation of pure Co [4,5]. The peaks in the XRD patterns of the pure Co samples after HPT are slightly shifted to lower diffraction angles. On the contrary, the Co75Cu25-L sample consists of ε Co and fcc Cu in the as-fabricated condition as expected. Both set of peaks are clearly distinguishable in the XRD pattern. Even in the as-fabricated condition, peaks of ε Co phase are shifted to lower diffraction angles and peaks of fcc Cu are slightly shifted to higher angles. After HPT deformation (as-deformed condition), only peaks of Co can be detected. Contrary to the HPT processed pure and doped Co samples, the α Co phase is now detected in the XRD pattern. This observation does not coincide with the equilibrium ε Co phase, which occur below temperatures of 695 K according to literature [6,28]. The influence of grain size on the obtained crystal structures in Co and Co-based alloys after HPT has been investigated in detail in [4,5,29,30]. Refining the grain size below 100 nm leads to an ε to α phase transformation with nanotwins and a minor amount of nanograins with other crystal structures (ε Co and distorted hcp Co) [5]. As a possible mechanism for the formation of the non-equilibrium α phase in Co for grain sizes below 100 nm during HPT deformation at room temperature, the continuous accumulation of stacking faults due to a deformation mechanism, which is mainly controlled by slip of partial dislocation in this grain size regime, is suggested [31]. In the XRD pattern in Fig. 4, peaks of the fcc Cu phase completely vanished after HPT deformation. Furthermore, the peak position of α Co is slightly shifted to lower diffraction angles after HPT.

The Co–Cu system has a positive heat of mixing and is immiscible in equilibrium at ambient temperatures [16.]. With different non-equilibrium processing methods like mechanical alloying the possibility to extend the mutual solubility of Co and Cu has already been reported [17–21]. The occurrence of supersaturated solid solutions and solid solubility levels is often determined from lattice parameter value changes given by the shifts in peak positions in the XRD patterns. SPD introduces stacking faults on (111) planes in fcc metals and on basal (0001) or prismatic (1010) planes of hcp metals which can cause anomalous peak broadening [32]. The stacking fault energy for pure fcc and hcp Co is 18.5 and 31 mJ/mm², respectively [33]. According to [5], a high density of stacking faults after HPT is induced in the material. Therefore, the contribution of stacking faults to broadening and shift of peak positions might be pronounced in Co due to its low stacking fault energy. For example, such an effect was shown in Cu–Co alloys processed by mechanical alloying [34]. Peak shifts due to stacking faults and peak shifts due to the extension of solid solubility can simultaneously occur further complicating the situation. The lattice parameter of the α Co phase determined from the peak positions in the XRD pattern in the as-deformed condition is 0.3568 nm. According to [35], this lattice parameter value corresponds to nearly 20 at% Cu which is dissolved in the α Co phase. Based on the XRD pattern and the SAD pattern of the as-deformed Co75Cu25 sample in Figs. 3c and 4, the formation of a single phase structure and a supersaturated solid solution of Cu in Co is suggested. Further investigations about the atomic scale distribution of Cu in Co by, e.g., atom probe tomography would be certainly necessary to give an exact extent of alloying.

### 3.2 Evaluation of thermal stability of the different nanostructures

**Fig. 5:**
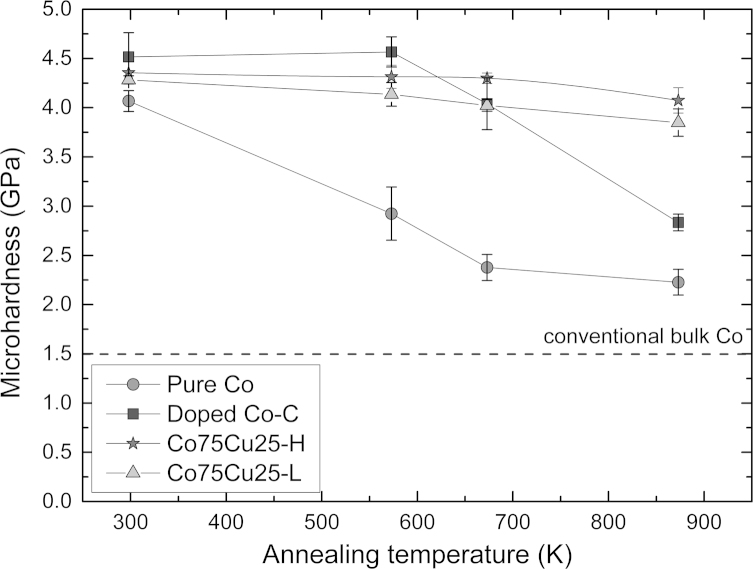
Evolution of the microhardness of the samples (pure Co, doped Co–C, Co75Cu25-L, Co75Cu25-H) as a function of the annealing temperature. The microhardness of the as-deformed samples is also included (values at a temperature of  293K, which corresponds to room temperature). Reference value for conventional Co is shown as broken line [6].

To study the thermal stability, the as-deformed samples are annealed at three different temperatures (573 K, 673 K and 873 K) for 1 h and changes in microhardness were measured subsequently. Microhardness as a function of the annealing temperature is plotted in Fig. 5. The microhardness of the pure Co sample rapidly drops even at the lowest annealing temperature of 573 K. Subsequently, the microhardness continuously decreases with increasing annealing temperature. The microhardness at the highest annealing temperature still exceeds the microhardness of conventional undeformed Co (HV~140 to 160 Nmm^−^²) [6]. The microhardness of the doped Co–C sample displays two distinct stages during the annealing treatment: the microhardness stays nearly constant till to annealing at 573 K. At higher annealing temperatures, the microhardness continuously drops indicating grain growth of the nanostructure. Finally, a microhardness of 2.38±0.08 GPa after annealing at 873 K for 1 h is obtained.

**Fig. 6:**
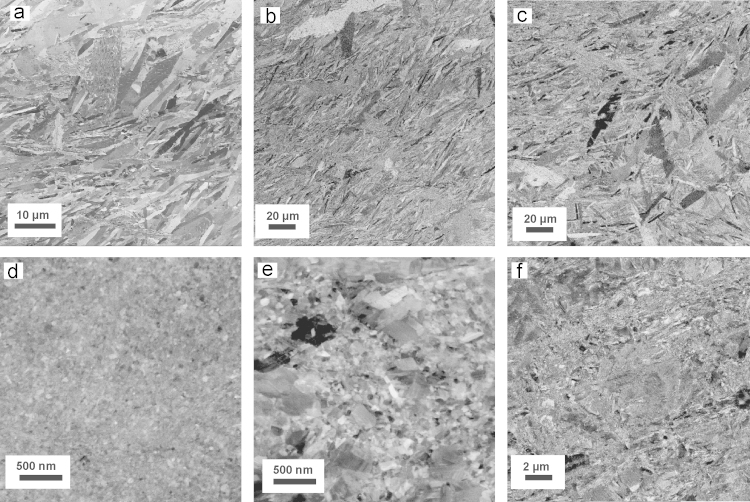
SEM images (back scattered electron mode) of the annealed microstructure of the pure Co sample after annealing for 1 h at (a) 573 K, (b) 673 K and (c) 873 K and the annealed microstructure of the doped Co–C sample after annealing for 1 h at (d) 573 K, (e) 673 K and (f) 873 K. All micrographs are recorded in tangential direction at a radius of 3.5 mm.

**Fig. 7:**
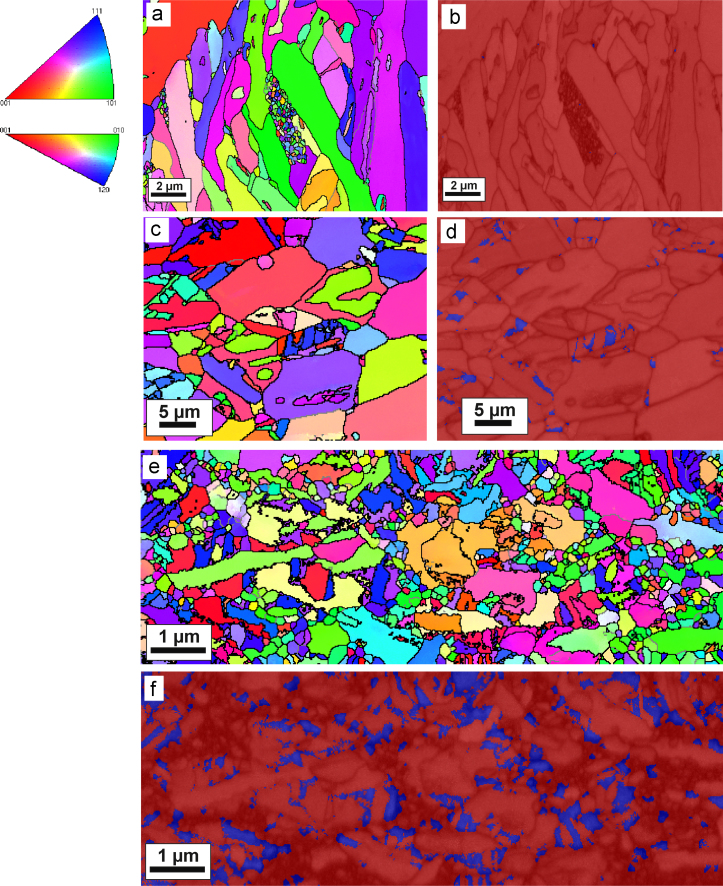
EBSD scans of the annealed microstructures. The standard triangles used for the EBSD scans is given on the left side. (a) Inverse pole figure map of pure Co after annealing at 573 K for 1 h. Low-angle grain boundaries (5°<*ω*<15°) are shown in gray and high-angle grain boundaries (*ω*>15°) and phase boundaries are shown in black in the micrograph (also valid for c and e). (b) The same EBSD scan, with all grains belonging to the ε Co are marked as red and all grains belonging to α Co are marked as blue. The EBSD scan is superimposed by an image quality map (band contrast): regions of low image quality are dark which correspond to unrecrystallized areas, while regions of high image quality (recrystallized grains) appear brighter (also valid for Fig. 7d and f). (c) Inverse pole figure map of pure Co after annealing at 873 K for 1 h. (d) Phase analysis map of the same area of doped Co after annealing at 873 K for 1 h. (e) Inverse pole figure map of doped Co–C after annealing at 673 K for 1 h. (f) Phase analysis map of doped Co–C after annealing at 673 K for 1 h the same area.

The microstructure of pure Co and doped Co–C samples after annealing at all three annealing temperatures is investigated by SEM. As displayed in Fig. 6a, the microstructure of the HPT deformed pure Co sample after annealing at 573 K for 1 h consists of two different regions: ultrafine grains are incorporated within a matrix of large, needle-shaped grains. This non-uniform microstructure is not observed at higher annealing temperatures any more. During annealing at 673 K, the ultrafine grained regions completely vanish and massive grain growth occurs (Fig. 6b). A uniform microstructure, consisting of large, needle shaped grains is visible, which is assumed to be the martensitic ε phase. Once the ultrafine grained region has been consumed, continuous growth of the microstructure proceeds and the mean size of the structural elements increases further at higher annealing temperatures. Some very large grains can be detected in the micrographs after annealing at 673 K and 873 K, too (Fig. 6b and c). An EBSD scan of the pure Co sample after annealing at 573 K is shown in Fig. 7a and b. In the inverse pole figure map (Fig. 7a), the same bimodal distribution of grain sizes, which is visible in the back scattered electron images, is observed. An EBSD phase analysis map of the same area, which is superimposed by a band contrast map, shows, that the pure Co sample predominately consists of ε (hcp) Co (Fig.7b). This observation is further confirmed by EBSD scans with larger scan sizes (~45×35 µm²). From these scans, an average amount of α Co below 0.05 area% is detected. To distinguish between the recrystallized parts of the annealed microstructure from the unrecrystallized parts, the so-called pattern quality map (band contrast) can be used in the EBSD analysis software. The band contrast is said to be closely related to the image quality of the EBSD scans. Bright grains (i.e. grains with a high average image quality) are considered as recrystallized grains and unrecrystallized grains appear dark (i.e. grains with low image quality). Regions with very large as well as very small grains occur in the microstructure of the EBSD scan (Fig. 7a). Considering the band contrast, the large grains in the EBSD scan appear brighter and are considered to be recrystallized ones. It is also possible to use orientations gradients in a grain to distinguish between recrystallized and unrecrystallized parts of the microstructure. Orientation gradients (different shades of the same color) are not visible in the large grains, which is a further indication for a recrystallized structure. In Fig. 7c, the inverse pole figure map of the pure Co sample after annealing at 873 K is shown. At higher annealing temperatures (673 K and 873 K), slightly higher amounts of α Co phase can be detected in the EBSD scans. For example, the average amount of α Co is only about 2 area% even after annealing at 873 K for 1 h (Fig. 7d). Considering the information, which can be gained by the superimposed band contrast and the orientation spread inside the grains, the annealed microstructure is fully recrystallized. Furthermore, no ultrafine grained regions can be detected in the EBSD scan any more.

The microstructural evolution during annealing of the doped Co–C sample is shown in Fig. 6d–f. In these samples, the annealing behavior is similar although two distinct differences occur: after annealing at 573 K, no difference between the as-deformed and annealed microstructure can be detected (Fig. 6d). Contrary to the pure Co sample at the same annealing temperature, the nc structure is still maintained. Although recovery processes might have already taken place, the grain size is not altered. After annealing at 673 K, abnormal grain growth sets in and the same bimodal type of microstructure as in pure Co after annealing at 573 K develops. However, there are some major differences: the grain shape of the large grains is rather globular and the size of the microstructural features is significantly smaller compared to the size in the abnormal grain growth region in the pure Co sample. At the highest annealing temperatures (873 K), further grain growth in the doped Co–C sample occurs, totally consuming the nc matrix (Fig. 6f). Nevertheless, the microstructure after annealing at this temperature is significantly smaller compared to the microstructure of the pure Co sample after annealing at the same temperature (Fig. 6c). Very large grains are also not observed in the annealed microstructure.

In Fig. 7e, the inverse pole figure map of the doped Co–C sample after annealing at 673 K and an EBSD phase analysis map of the same area, which is superimposed by the band contrast, is illustrated. In the inverse pole figure map, the dual-sized microstructure is also observed. Large grains are located next to regions of very small grains. The orientation spread inside the large grains is small and their image quality is higher compared to the fine grained region. Therefore, large grains in the EBSD scan can be considered as recrystallized. Surprisingly, the EBSD phase analysis clearly shows that the doped Co–C sample exhibits a dual-phase structure consisting of α and ε Co phases. The α phase amount is nearly 20 area%. Contrary, only 2 area% of α phase is detected in the pure Co sample even after annealing at the highest annealing temperature. The fine grained regions in the EBSD scan predominately consist of ε Co. According to literature, the reversible martensitic phase change occurs at ~695 K in equilibrium [6,28]

**Fig. 8:**
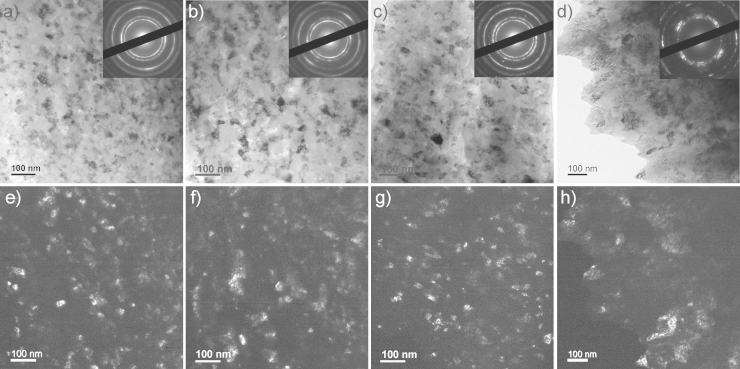
TEM bright and dark field images with corresponding SAD patterns (see insets) showing the microstructure of the Co75Cu25-L sample in the as-deformed condition (a and e) and after annealing for 1 h at 573 K (b and f), 673 K (c and g) and 873 K (d and f).

**Fig. 9:**
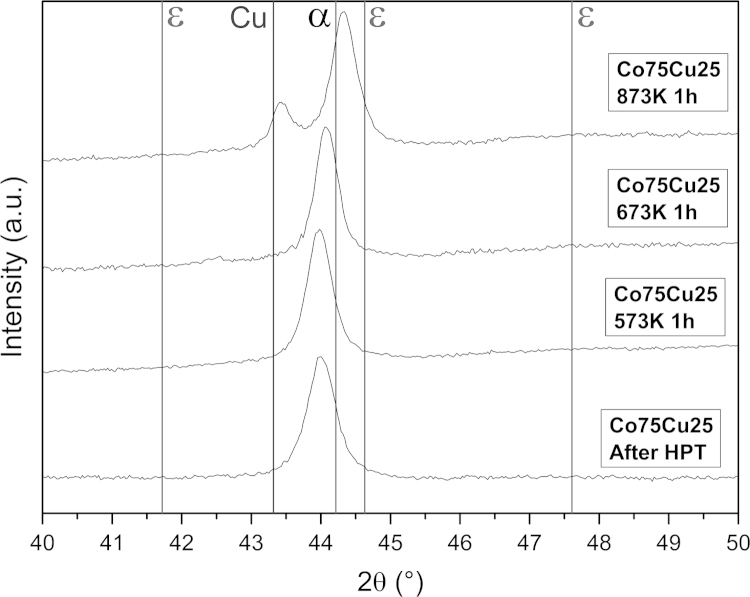
XRD patterns of the Co75Cu25-L sample recorded in the as deformed condition (denoted as ‘After HPT’) and after annealing for 1 h at 573 K, 673 K and 873 K. Peaks of hcp Co (ε), fcc Co (α) and fcc Cu (Cu) are indicated in the plots.

The microhardness as a function of the annealing temperature of the Co75Cu25-L samples exhibits a completely different behavior compared to the pure Co samples: it stays nearly constant at low annealing temperatures of 573 K and 673 K (Fig. 5). At the highest annealing temperature of 873 K, the measured microhardness value slightly decreases, but it is still in the range of the doped Co–C sample annealed for 1 h at 673 K. It is furthermore significantly enhanced compared to the reference value of conventional Co (HV~140 to 160 Nmm^−^²) [6]. Not only the evolution of the microhardness as a function of annealing temperature, also the microstructural evolution during annealing in the Co75Cu25-L samples is different compared to the pure Co samples. In Fig. 8, the microstructures after annealing at different annealing temperatures are shown in bright and dark field TEM images. Compared to the as-deformed microstructure, the grain size is not significantly changed after annealing at 573 K and 673 K (see dark field images in Fig. 8e–g). In both samples an apparent homogeneous microstructure is still visible. The corresponding SAD patterns (see insets in Fig. 8a–c) reflect the nc structure as well. In the as-deformed condition and after annealing at 573 K, only fcc Co Debye–Scherrer rings of (111), (200), (220), (311) and (222) are visible. After annealing at 673 K, the SAD pattern shows the fcc Co Debye–Scherrer rings as well. Additionally, single spots of the hcp Co phase are visible (see inset in Fig. 8c). In the TEM micrographs of the sample annealed at 873 K, a somewhat larger grain size is visible (Fig. 8h). The SAD pattern of the sample shows two sets of spotty diffraction rings including the fcc Cu and hcp Co phase (see inset Fig. 8d).Conventional EBSD in the SEM is still limited by the pattern source volume and it is rather difficult to perform for truly nc materials [36]. XRD investigations of the Co75Cu25-L samples in the annealed conditions were performed as well. In Fig. 9, XRD patterns after annealing and in the as-deformed condition as a reference (indicated as ‘After HPT’ in the plot) are shown. According to the XRD data, no phase changes could be observed after annealing at 573 K. Still, only α Co is detected and the peak position is nearly unchanged. After annealing at 673 K, the α Co peak is shifted to somewhat higher diffraction angles, but no fcc Cu phase peak or ε Co peaks are detected in the XRD pattern. The lattice parameter of α Co decreases from 0.3568 nm (as-deformed), to 0.3561 nm (573 K) and to 0.3557 nm after annealing at 673 K. Based on this observation, decomposition of the single phase structure after annealing at 573 K and 673 K already starts. According to [35], about 15 at% and 9 at% Cu are still dissolved in Co after annealing at 573 K and 673 K, respectively. After annealing at 873 K, the fcc Cu phase peak appears again in the XRD pattern, which is another indication for the decomposition of the single phase structure at enhanced temperatures.

**Fig. 10:**
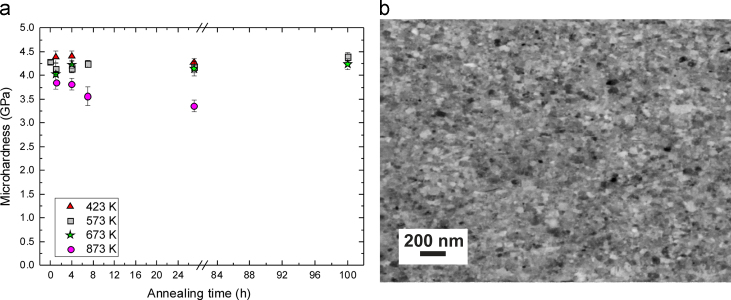
(a) Evolution of the microhardness of the Co75Cu25-L sample annealed at 423 K, 573 K, 673 K, and 873 K as a function of the annealing time. The microhardness of the as-deformed samples is also included in the plot (values at annealing time of 0 h). (b) SEM images (back scattered electron mode) illustrating the microstructure of the Co75Cu25-L sample annealed at 673 K for 100 h.

The influence of long term annealing (annealing time of several hours) on the microhardness and microstructural evolution of the Co75Cu25-L sample, which is chosen due to its enhanced thermal stability in the previous ‘short time’ annealing experiments, is furthermore investigated. As-deformed Co75Cu25-L samples are annealed at four different temperatures (423 K, 573 K, 673 K and 873 K) for 1 h up to 100 h and the evolution of the microhardness as a function of the annealing time is shown in Fig. 10a. At low annealing temperatures (423 K–673 K), nearly no change in the microhardness can be detected even after annealing for very long times (up to 100 h). Upon annealing at the highest annealing temperature (873 K), the microhardness is continuously decreasing with increasing annealing time which goes hand in hand with minor structural coarsening. After annealing for 27 h at 873 K, a microhardness of 3.36±0.12 GPa is measured, still significantly higher compared to conventional Co [6]. For annealing at 423 K, 573 K and 673 K for different times, no significant change or coarsening of the microstructure takes place. To give an example for the microstructural evolution during the long term annealing experiments, a micrograph of the Co75Cu25-L sample after annealing for 100 h at 673 K is illustrated in Fig. 10b.

## 4 Comparison of the different strategies to enhance the thermal stability of nc materials

### 4.1 The effect of carbon on thermal stability

Each pure Co sample undergoes an initial sequence of abnormal grain growth at low annealing temperatures, in which large grains grow into a still ultrafine grained matrix. In the pure Co sample doped with carbon, the starting point of abnormal grain growth occurs at slightly higher annealing temperatures (673 K compared to 573 K in the pure Co sample) and results in microstructures with an overall smaller size even after massive grain growth in the structure occurred.

The total amount of impurity elements is the same in both Co materials and both are processed and annealed under exactly the same conditions (Table 1). As listed in Table 1, the sulfur-content in our Co samples (pure Co and doped Co–C) is very low (1 ppm). Sulfur, unlike as in ED processed Co, cannot be responsible for the observed enhanced thermal stability in the carbon doped Co samples. The role of oxides on the enhancement of thermal stability of the nanostructures, especially since oxygen is included in a relatively high amount (351 ppm) in the pure Co samples (Table 1), can be considered as well but is easily ruled out. Again, the amount of oxygen is the same in both Co materials. Hence, the carbon content is the only difference and an influence of carbon on the thermal stability at least at low annealing temperatures is evident. At higher annealing temperatures, significant grain growth and the complete loss of the nanostructure is observed in the carbon doped samples as well. This raises the question why adding small amounts of carbon can impede grain growth at low annealing temperatures, but obviously the effect is lost at higher annealing temperatures?

Grain growth occurs due to migration and coalescence of grain boundaries [37]. Reducing the grain boundary energy, and, as a consequence, the driving force for grain growth enhances thermal stability. From a thermodynamic point of view, solutes or impurity elements, which segregate to the grain boundaries, will lower their energy [38–41]. Furthermore, kinetic contributions based on solute drag or other dragging forces due to pores, triple junctions or small second phase particles can exist [42–44]. The velocity of a grain boundary (*v*_*GB*_) is given as the product of the driving force *P* and grain boundary mobility *m*_*GB*_ [45]:

(1) $$v_{GB}=Pm_{GB}$$

For solute or impurity segregation to grain boundaries, the grain boundary mobility is lowered [45]. Suitable solutes are conventionally chosen by estimating solute segregation strength based on large atomic size mismatch with the solvent or low bulk solid solubility [46–50]. In [46,50], it is shown that the relative amount of segregation to grain boundaries is indeed inversely proportional to the solubility limit of the solute or impurity element. The initial, as deformed crystal structure of the carbon doped sample is ε Co. In ε Co, the solubility of carbon is very low (8.74×10^−4^ at% at the eutectoid temperature of 694.5 K) [46,50]. Therefore, segregation of carbon atoms to grain boundaries is very likely. The possibility of carbon segregation to grain boundaries is also dependent on the mobility of the solute or impurities (i.e. carbon atoms) during annealing. The mobility can be estimated by the average diffusion distance *x* using the random walk theory of diffusion [51]

(2) $$x=\sqrt{Dt}$$

where *D* is the diffusion coefficient and *t* is the time. The carbon diffusion coefficient *D* can be estimated by extrapolating the Arrhenius equation to lower temperatures [52]

(3) $$D=D_{0}\;\exp\left(\frac{-Q}{k_{B}T}\right)$$

For carbon in Co, values of *D*_0_=0.0872 cm² s^−1^ and *Q*=149.3 kJ mol^−1^ (temperature range 723–1073 K) can be found in literature [53]. For a temperature of 573 K, a diffusion coefficient *D*_573 K_~2×10^−15^ cm² s^−1^ is obtained. It is now possible to estimate the average diffusion distance of carbon at 573 K (*x*_573 K_ ) using *D*_573 K_ and a time *t* of 3600 s (corresponding to the annealing time of 1 h), which yields a *x*_573K_ of ~28 nm. The carbon atoms have at least to diffuse over a distance *x*~*d*/4, if *d* is the mean grain size of the material. For a typical grain size of *d*≈100 nm, which is definitely the upper limit of the grain size in the carbon doped material (see Figs. 3b and 6d), the carbon atoms have to diffuse over a distance of 25 nm. Additionally, athermally produced excess vacancies are present in high concentrations after SPD accelerating diffusion processes [54,55]. It seems realistic to assume, that even during annealing at the lowest annealing temperature (573 K), carbon can easily migrate to the grain boundaries in the doped Co–C sample and stabilize the nanostructure. In fact, 3D-atom probe tomography of nc Ni, which has a similar limited solubility of carbon and a comparable grain size in the as-deformed condition without annealing, has shown that carbon atoms are mainly located at grain boundaries even at room temperature [14,56]. Therefore, grain growth or growth inhibition for annealing at low temperatures seems to be mainly solute/impurity drag controlled in the carbon-doped Co material. In the pure Co samples, which has a very low amount of impurities, a significant fraction of grains has already grown abnormally in the ultrafine grained matrix at the same annealing temperature.

For annealing at 673 K, abnormal grain growth is also observed in the carbon doped samples. Phase transformation (from the martensitic ε phase to the fcc α phase) already occurs for annealing at 673 K which is lower as expected from literature values of the equilibrium phase transformation temperature of pure Co [6]. The microstructure after annealing at 673 K consists of a mixture of ~80 area% ε Co and ~20 area% α Co at room temperature. During annealing at 673 K, the amount of α Co is supposed to be even higher due to the *α*→*ε* phase transformation which in turn occurs during cooling. Furthermore, phase transformation from the ε to α phase and onset of abnormal grain growth seems to be in the same temperature range in the doped Co–C samples.

The temperature of the allotropic phase transformation of Co is sensitive to experimental conditions (i.e. rate of temperature change) and depends additionally on the purity of the material. Carbon, among some other alloying elements, suppress the transformation temperature and is known as fcc stabilizer for Co [46,57]. The metastable fcc phase can also be stabilized by a small grain size at room temperature. According to [57], even in commercial cobalt, which is deformed, subsequently annealed and cooled down to room temperature, the fcc phase can be found besides the equilibrium hcp phase at room temperature. Consequently, the allotropic phase transformation might affect the microstructural evolution during annealing as well. Although the solubility of carbon in ε Co is very low, carbon has a rather good solubility in α Co (almost 2 at% at 1173 K) [50]. Furthermore, the magnitude of segregation of solute or impurity elements to grain boundaries is inversely proportional to their solubility limit [46,50]. Due to the higher solubility of carbon in α Co, it is assumed, that the carbon concentration at the grain boundaries is lowered due to the beginning allotropic phase transformation from ε to α Co. The mobility of a grain boundary, which has to move together with segregated solutes or impurities, is defined as [45]

(4) $$m_{GB\;}=\frac{D}{\Gamma k_{B}T}$$

where *D* is the diffusion coefficient of the solute/impurity element, *Γ* is the grain boundary absorption factor and *T* is the temperature. For annealing at higher temperatures, *Γ* is decreased due to dissolution of carbon in the matrix and the carbon diffusion coefficient is significantly increased (*D*_673 K_~2×10^−13^ cm² s^−1^ and *D*_873 K_~1×10^−10^ cm² s^−1^). Hence, a migrating grain boundary has now to drag a substantial lower amount of carbon and its mobility is increased according to Eq. (4). Due to the successive ε to a α transformation of the grains, carbon segregation at grain boundaries is inhomogenously distributed, which induces the start of abnormal grain growth (Fig. 7e and f). Comparing the annealed microstructures of carbon doped Co–C to pure Co samples, growth appears to be more uniformly and grain size changes in total are lower (from below 100 nm to a size below ~1 µm). Once growth has started in pure Co, the grains size changes from ~100 nm to ~20 µm (from the largest grains in Fig. 6b), which is a change in the linear dimension by a factor of 200. Substantial driving force for grain growth is maintained due to the non-uniform microstructure in pure Co (i.e. larger grains shown in Fig. 6b and c). Hence, addition of small amounts of carbon has a positive pinning effect even at higher annealing temperatures.

Another type of dragging force must be considered in the carbon doped samples. In the equilibrium Co–C system, cooling of a solid solution from high temperatures to room temperature results in phase transformation from α to ε Co with graphite precipitations [46]. However, no peaks of graphite precipitates are observed in the XRD pattern of the carbon doped sample in the as-deformed condition. They may still form, but cannot be detected due to the very low total amount of carbon (~500 ppm) in the sample.

#### 4.2 The effect of Cu alloying on thermal stability

Processing Co–Cu powder mixtures by HPT and alloying Co with 25 wt% Cu results in a substantial enhancement of the thermal stability of the as-deformed nanostructures, which remain stable even after very long annealing times (Fig. 10a and b). Furthermore, abnormal grain growth is not observed in the alloyed Co samples. Long term-annealing below 673 K results in a stabilized nanostructure instead, without any significant amount of grain growth. Only at the highest annealing temperature (873 K), minor grain coarsening occurs and grain growth slowly continues with ongoing annealing time. Even then, a uniform microstructure without abnormally grown grains is maintained. This is a significant improvement compared to previous studies, which investigated the thermal stability of ED synthesized nc materials. For example, the thermal stability of nc Co alloyed with Ni prepared by ED reports significant grain growth during annealing for 7200 s at 573 K [10].

Several factors may contribute to the enhanced thermal stability of the alloyed samples. Compacted micrometer sized powder samples are used as starting material, which are subsequently HPT deformed. Contaminates from the raw powder material itself are introduced in the latter bulk material during processing which can enhance the thermal stability of the material. However, Co75Cu25-H samples, processed of Co and Cu powders with a higher purity and additionally compacted and sintered under vacuum conditions, exhibit identical thermal stability. Neither a significant difference in the microstructure in the as-deformed condition, nor in the annealed microstructures or in the evolution of the microhardness as a function of the annealing temperature can be observed. The influence of impurities might therefore be less important on the thermal stability of this type of nanostructures. Alternative grain boundary pinning mechanism can originate from small oxide particles. During HPT processing of powders, oxides from the natural oxide layer of the powder particles are incorporated, fragmented and homogenously distributed in the metallic matrix. For example, an excellent thermal stability in nc Ni processed by HPT compaction and deformation of Ni powders due to nanometer sized and finely dispersed NiO particles is achieved [15,58]. Contrary to [15,58], in which the thickness of the natural oxide layer on the Ni powder particles was artificially increased by annealing prior to compaction leading to about 10 vol% NiO in the material, no oxide peaks are detected in the XRD patterns of the alloyed Co samples. However, the role of oxides on the enhanced thermal stability cannot be entirely excluded.

In addition, the formation of single phase α Co supersaturated solid solutions is supposed to occur in the as-deformed condition based on XRD investigations. In literature, the α Co phase is reported to be the stable phase if a certain amount of Cu is dissolved in Co, which is a further indication for the formation of supersaturated solid solutions in this system. During annealing, phase separation is thermodynamically favored and decomposition of the single phase structure is expected. The lattice parameter of α Co determined from the peak position in the XRD patterns continuously decreases after annealing at 573 K and 673 K indicating that decomposition into a two phase structure has already started at this annealing temperatures. This decomposition process proceeds during annealing at higher temperatures, since fcc Cu as well as hcp Co peaks become clearly visible in the XRD and SAD pattern after annealing at 873 K. After complete decomposition, a two phase structure is obtained in the material, in which separated Co and Cu phases exist next to each other. Grain growth through boundary migration might now be lowered due to this structural configuration (grains of the one phase may be isolated from the other phase). Another mechanism which can be partially responsible for the remarkable thermal stability is the decrease of effective grain boundary free energy due to Cu segregation in the grain boundaries.

**Fig. 11:**
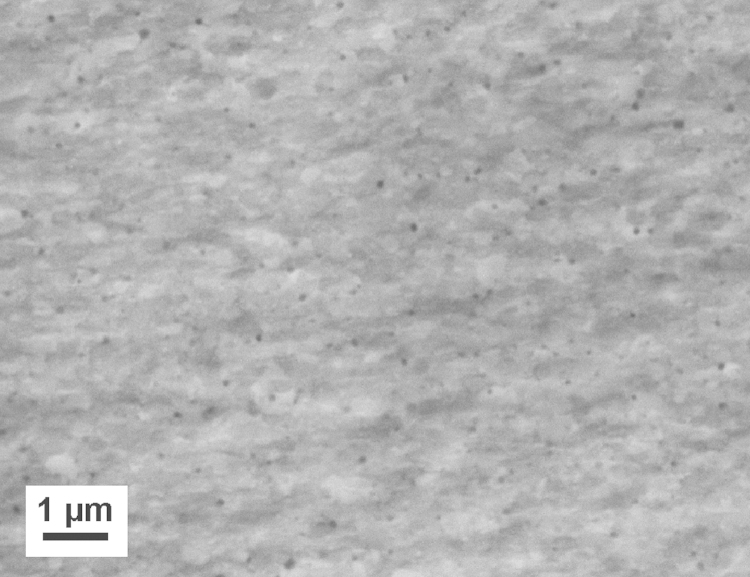
SEM image (secondary electron mode) of the microstructure of the Co75Cu25-L sample annealed at 873 K for 27 h showing a large amount of small pores.

But there is one more important aspect, which must finally be considered in the discussion on the enhanced thermal stability of the Co–Cu powder mixtures processed by HPT. Fig. 11 shows the microstructure of the Co75Cu25-L sample after annealing at the highest annealing temperature (873 K) for the longest annealing time (27 h) at this annealing temperature. As already mentioned in the result section, some grain growth occurred leading to grains now in the ultrafine grained range. But more important is another interesting feature, which becomes visible in the structure due to grain growth. A large number of small pores, which are mainly located at grain boundaries and triple junctions, are now clearly visible.

Even nc materials, which are considered to be fully dense and synthesized by different techniques (ED, HPT and inert gas condensation), contain 1–2 nm sized pores and even smaller nanovoids [59]. In our samples, the content might be even higher due to powder compaction before HPT deformation. Nanovoids or small pores can hinder the motion of grain boundaries similar to second phase particles as recently shown in [60]. Material strengthening and grain growth inhibition by voids and pores have also been observed, for example, in [61–64]. In the as-deformed and annealed states (at lower annealing temperatures without significant grain growth), the pores are not easily detected in the micrographs due to their nanometer size, which is always smaller than the grain size at every stage of microstructural evolution.

Future work will concentrate on mechanical properties of the processed nanostructures. Promising results from [14], no loss of ductility in carbon doped nc Ni samples, and from [65], at least some ductility in cobalt samples containing nanovoids, are already reported. Hence, the combination of high-strength nc materials with reasonable ductility and high thermal stability together with their extraordinary properties for technological applications like MEMS might be possible.

## 5 Conclusions

Uniform ultrafine grained microstructures and true nanostructures can be produced by HPT of pure Co and pure Co doped with small amounts of carbon and HPT of Co–Cu powder mixtures. Both investigated pure Co materials consist of ε-Co structure, the Co–Cu alloy material exhibits a α phase structure. Doping pure Co with small amounts of carbon or alloying with 25 wt% Cu reduces the saturation grain size and increases the microhardness. During the evaluation of the thermal stability, two different types of annealed microstructures are observed. The first type, obtained during isothermal annealing of pure Co and carbon doped Co samples at low temperatures, is of a non-uniform grain structure with large grains embedded in an ultrafine grained or nc matrix. During annealing at higher temperatures, significant grain growth occurs in both structures. Due to the addition of carbon, a shift of the temperature of abnormal grain growth (~100 K) can be obtained. Initiation of abnormal grain growth is linked to dissolution of carbon in α Co during partial allotropic phase transformation of the structure and reduction of impurity/solute concentration at the grain boundaries. Grain growth furthermore occurs at a slower rate and more homogenously due to the addition of carbon.

A substantial increase in thermal stability is achieved by HPT deformation of Co–Cu powder compacts and a completely different type of annealed microstructure develops. The nanostructure stays uniform and unaltered for isothermal annealing at temperatures below 673 K even after annealing for very long times. Apart from minor grain growth, structural stability is further maintained for annealing at 873 K longer than one day. Among the enhanced stability due to the formation of a single phase α Co supersaturated solid solution and subsequent phase separation, a very efficient way to stabilize nanostructures might be a homogeneous distribution of nanometer sized pores. These pores have always a size smaller than the grain size in the corresponding material.

The following main conclusions can be drawn from the present work: by circumventing abnormal grain growth in nanostructures, the thermal stability can be significantly improved. This aim can also be achieved by introducing nanometer sized pores in the structure. Grain boundary pinning by impurity/solute segregation (solute drag) hinder grain boundary migration to a certain extent, but are less effective.

## Figures and Tables

**Table 1:** Purity of Co materials used in this study. Only impurity elements with values ≥0.1 ppm are listed.

Material	Co (wt%)	Total impurity content (ppm)	Carbon content (ppm)	Impurity elements (ppm)
Pure Co	99.96	395	8	Cu(0.1), Mg(0.12), N(2), Ni(18), O(351), S(1), Cr (0.22), Fe(12), H(<1), P(0.1), Si(0.1), Ta(<1)
Doped Co–C	99.92	844	457	Same as above

**Table 2:** Microhardness in the as-deformed and annealed conditions, and crystal structures before and after annealing for pure Co, doped Co–C, Co75Cu25-L and Co75Cu25-H samples.

	Pure Co	Doped Co–C	Co75Cu25 L	Co75Cu25 H
As deformed phases	hcp Co	hcp Co	fcc Co	fcc Co
Microhardness (GPa)	4.07±0.11	4.52±0.25	4.28±0.04	4.35±0.11
As-annealed phases (573 K 1 h)	hcp Co	–	fcc Co	–
Microhardness (573 K 1 h)	2.23±0.17	4.57±0.15	4.14±0.12	4.31±0.12
As-annealed phases (673 K 1 h)	hcp Co	hcp+fcc Co	fcc Co+hcp Co	–
Microhardness (673 K 1 h)	2.18±0.13	4.04±0.26	4.02±0.06	4.29±0.05
As-annealed phases (873 K 1 h)	hcp+fcc Co	hcp+fcc Co	hcp Co+fcc Cu	–
Microhardness (873 K 1 h)	2.23±0.13	2.38±0.08	3.85±0.14	4.07±0.13
